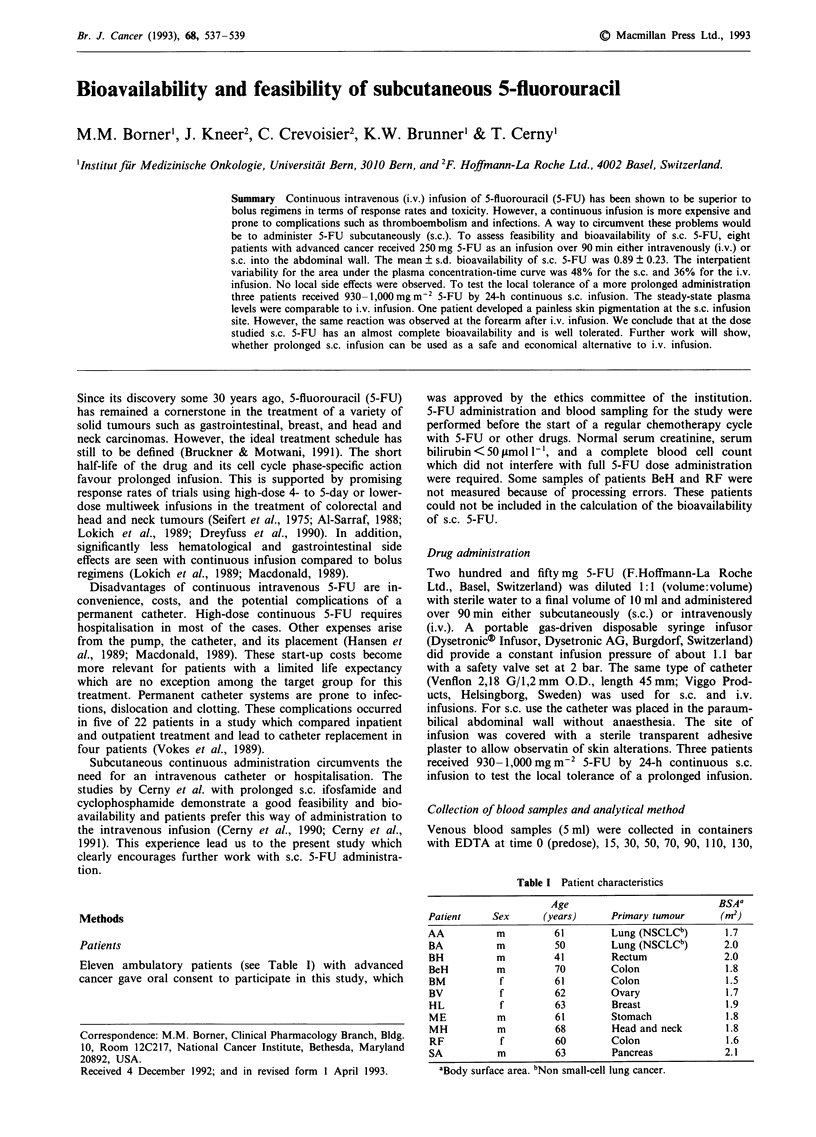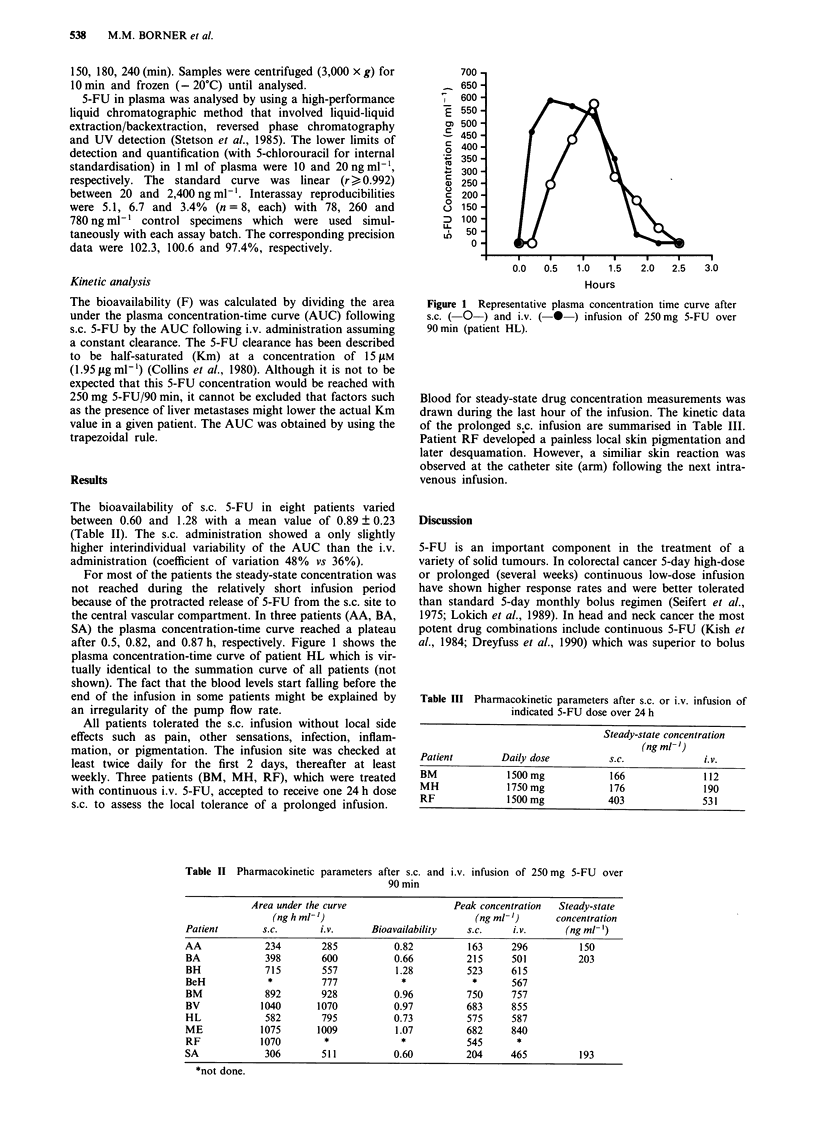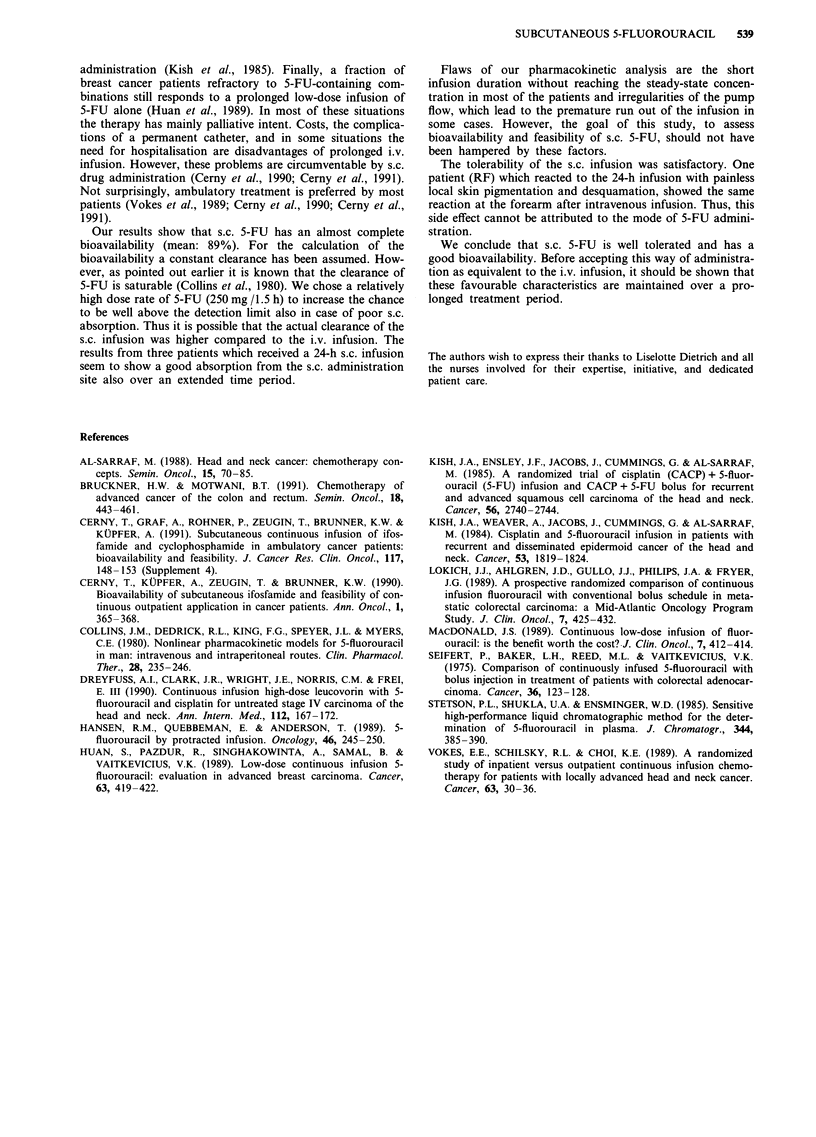# Bioavailability and feasibility of subcutaneous 5-fluorouracil.

**DOI:** 10.1038/bjc.1993.382

**Published:** 1993-09

**Authors:** M. M. Borner, J. Kneer, C. Crevoisier, K. W. Brunner, T. Cerny

**Affiliations:** Institut für Medizinische Onkologie, Universität Bern, Switzerland.

## Abstract

Continuous intravenous (i.v.) infusion of 5-fluorouracil (5-FU) has been shown to be superior to bolus regimens in terms of response rates and toxicity. However, a continuous infusion is more expensive and prone to complications such as thromboembolism and infections. A way to circumvent these problems would be to administer 5-FU subcutaneously (s.c.). To assess feasibility and bioavailability of s.c. 5-FU, eight patients with advanced cancer received 250 mg 5-FU as an infusion over 90 min either intravenously (i.v.) or s.c. into the abdominal wall. The mean +/- s.d. bioavailability of s.c. 5-FU was 0.89 +/- 0.23. The interpatient variability for the area under the plasma concentration-time curve was 48% for the s.c. and 36% for the i.v. infusion. No local side effects were observed. To test the local tolerance of a more prolonged administration three patients received 930-1,000 mg m-2 5-FU by 24-h continuous s.c. infusion. The steady-state plasma levels were comparable to i.v. infusion. One patient developed a painless skin pigmentation at the s.c. infusion site. However, the same reaction was observed at the forearm after i.v. infusion. We conclude that at the dose studied s.c. 5-FU has an almost complete bioavailability and is well tolerated. Further work will show, whether prolonged s.c. infusion can be used as a safe and economical alternative to i.v. infusion.


*Br. J. Cancer* (1993), **68**, 537–539 © Macmillan Press Ltd., 1993

### Bioavailability and feasibility of subcutaneous 5-fluorouracil

M.M. Borner[1], J. Kneer[2], C. Crevoisier[2], K.W. Brunner[1] & T. Cerny[1]

[1]*Institut für Medizinische Onkologie, Universität Bern, 3010 Bern, and* [2]*F. Hoffmann-La Roche Ltd., 4002 Basel, Switzerland.*

**Summary** Continuous intravenous (i.v.) infusion of 5-fluorouracil (5-FU) has been shown to be superior to bolus regimens in terms of response rates and toxicity. However, a continuous infusion is more expensive and prone to complications such as thromboembolism and infections. A way to circumvent these problems would be to administer 5-FU subcutaneously (s.c.). To assess feasibility and bioavailability of s.c. 5-FU, eight patients with advanced cancer received 250 mg 5-FU as an infusion over 90 min either intravenously (i.v.) or s.c. into the abdominal wall. The mean ± s.d. bioavailability of s.c. 5-FU was 0.89 ± 0.23. The interpatient variability for the area under the plasma concentration-time curve was 48% for the s.c. and 36% for the i.v. infusion. No local side effects were observed. To test the local tolerance of a more prolonged administration three patients received 930–1,000 mg $m^{-2}$ 5-FU by 24-h continuous s.c. infusion. The steady-state plasma levels were comparable to i.v. infusion. One patient developed a painless skin pigmentation at the s.c. infusion site. However, the same reaction was observed at the forearm after i.v. infusion. We conclude that at the dose studied s.c. 5-FU has an almost complete bioavailability and is well tolerated. Further work will show, whether prolonged s.c. infusion can be used as a safe and economical alternative to i.v. infusion.

Since its discovery some 30 years ago, 5-fluorouracil (5-FU) has remained a cornerstone in the treatment of a variety of solid tumours such as gastrointestinal, breast, and head and neck carcinomas. However, the ideal treatment schedule has still to be defined (Bruckner & Motwani, 1991). The short half-life of the drug and its cell cycle phase-specific action favour prolonged infusion. This is supported by promising response rates of trials using high-dose 4- to 5-day or lower-dose multiweek infusions in the treatment of colorectal and head and neck tumours (Seifert *et al.*, 1975; Al-Sarraf, 1988; Lokich *et al.*, 1989; Dreyfuss *et al.*, 1990). In addition, significantly less hematological and gastrointestinal side effects are seen with continuous infusion compared to bolus regimens (Lokich *et al.*, 1989; Macdonald, 1989).

Disadvantages of continuous intravenous 5-FU are inconvenience, costs, and the potential complications of a permanent catheter. High-dose continuous 5-FU requires hospitalisation in most of the cases. Other expenses arise from the pump, the catheter, and its placement (Hansen *et al.*, 1989; Macdonald, 1989). These start-up costs become more relevant for patients with a limited life expectancy which are no exception among the target group for this treatment. Permanent catheter systems are prone to infections, dislocation and clotting. These complications occurred in five of 22 patients in a study which compared inpatient and outpatient treatment and lead to catheter replacement in four patients (Vokes *et al.*, 1989).

Subcutaneous continuous administration circumvents the need for an intravenous catheter or hospitalisation. The studies by Cerny *et al.* with prolonged s.c. ifosfamide and cyclophosphamide demonstrate a good feasibility and bioavailability and patients prefer this way of administration to the intravenous infusion (Cerny *et al.*, 1990; Cerny *et al.*, 1991). This experience lead us to the present study which clearly encourages further work with s.c. 5-FU administration.

### Methods

### *Patients*

Eleven ambulatory patients (see Table I) with advanced cancer gave oral consent to participate in this study, which was approved by the ethics committee of the institution. 5-FU administration and blood sampling for the study were performed before the start of a regular chemotherapy cycle with 5-FU or other drugs. Normal serum creatinine, serum bilirubin < 50 μmol $l^{-1}$, and a complete blood cell count which did not interfere with full 5-FU dose administration were required. Some samples of patients BeH and RF were not measured because of processing errors. These patients could not be included in the calculation of the bioavailability of s.c. 5-FU.

### *Drug administration*

Two hundred and fifty mg 5-FU (F.Hoffmann-La Roche Ltd., Basel, Switzerland) was diluted 1:1 (volume:volume) with sterile water to a final volume of 10 ml and administered over 90 min either subcutaneously (s.c.) or intravenously (i.v.). A portable gas-driven disposable syringe infusor (Dysetronic® Infusor, Dysetronic AG, Burgdorf, Switzerland) did provide a constant infusion pressure of about 1.1 bar with a safety valve set at 2 bar. The same type of catheter (Venflon 2,18 G/1,2 mm O.D., length 45 mm; Viggo Products, Helsingborg, Sweden) was used for s.c. and i.v. infusions. For s.c. use the catheter was placed in the paraumbilical abdominal wall without anaesthesia. The site of infusion was covered with a sterile transparent adhesive plaster to allow observatin of skin alterations. Three patients received 930–1,000 mg $m^{-2}$ 5-FU by 24-h continuous s.c. infusion to test the local tolerance of a prolonged infusion.

### *Collection of blood samples and analytical method*

Venous blood samples (5 ml) were collected in containers with EDTA at time 0 (predose), 15, 30, 50, 70, 90, 110, 130,

**Table I** Patient characteristics

| *Patient* | *Sex* | *Age (years)* | *Primary tumour* | *BSA[a] ($m^2$)* |
|---|---|---|---|---|
| AA | m | 61 | Lung (NSCLC[b]) | 1.7 |
| BA | m | 50 | Lung (NSCLC[b]) | 2.0 |
| BH | m | 41 | Rectum | 2.0 |
| BeH | m | 70 | Colon | 1.8 |
| BM | f | 61 | Colon | 1.5 |
| BV | f | 62 | Ovary | 1.7 |
| HL | f | 63 | Breast | 1.9 |
| ME | m | 61 | Stomach | 1.8 |
| MH | m | 68 | Head and neck | 1.8 |
| RF | f | 60 | Colon | 1.6 |
| SA | m | 63 | Pancreas | 2.1 |

[a]Body surface area. [b]Non small-cell lung cancer.

Correspondence: M.M. Borner, Clinical Pharmacology Branch, Bldg. 10, Room 12C217, National Cancer Institute, Bethesda, Maryland 20892, USA.
Received 4 December 1992; and in revised form 1 April 1993.

150, 180, 240 (min). Samples were centrifuged (3,000 × g) for 10 min and frozen (− 20°C) until analysed.

5-FU in plasma was analysed by using a high-performance liquid chromatographic method that involved liquid-liquid extraction/backextraction, reversed phase chromatography and UV detection (Stetson et al., 1985). The lower limits of detection and quantification (with 5-chlorouracil for internal standardisation) in 1 ml of plasma were 10 and 20 ng ml$^{-1}$, respectively. The standard curve was linear ($r \geqslant 0.992$) between 20 and 2,400 ng ml$^{-1}$. Interassay reproducibilities were 5.1, 6.7 and 3.4% ($n = 8$, each) with 78, 260 and 780 ng ml$^{-1}$ control specimens which were used simultaneously with each assay batch. The corresponding precision data were 102.3, 100.6 and 97.4%, respectively.

*Kinetic analysis*

The bioavailability (F) was calculated by dividing the area under the plasma concentration-time curve (AUC) following s.c. 5-FU by the AUC following i.v. administration assuming a constant clearance. The 5-FU clearance has been described to be half-saturated (Km) at a concentration of 15 μM (1.95 μg ml$^{-1}$) (Collins et al., 1980). Although it is not to be expected that this 5-FU concentration would be reached with 250 mg 5-FU/90 min, it cannot be excluded that factors such as the presence of liver metastases might lower the actual Km value in a given patient. The AUC was obtained by using the trapezoidal rule.

### Results

The bioavailability of s.c. 5-FU in eight patients varied between 0.60 and 1.28 with a mean value of 0.89 ± 0.23 (Table II). The s.c. administration showed a only slightly higher interindividual variability of the AUC than the i.v. administration (coefficient of variation 48% vs 36%).

For most of the patients the steady-state concentration was not reached during the relatively short infusion period because of the protracted release of 5-FU from the s.c. site to the central vascular compartment. In three patients (AA, BA, SA) the plasma concentration-time curve reached a plateau after 0.5, 0.82, and 0.87 h, respectively. Figure 1 shows the plasma concentration-time curve of patient HL which is virtually identical to the summation curve of all patients (not shown). The fact that the blood levels start falling before the end of the infusion in some patients might be explained by an irregularity of the pump flow rate.

All patients tolerated the s.c. infusion without local side effects such as pain, other sensations, infection, inflammation, or pigmentation. The infusion site was checked at least twice daily for the first 2 days, thereafter at least weekly. Three patients (BM, MH, RF), which were treated with continuous i.v. 5-FU, accepted to receive one 24 h dose s.c. to assess the local tolerance of a prolonged infusion.

**Figure 1** Representative plasma concentration time curve after s.c. (—O—) and i.v. (—●—) infusion of 250 mg 5-FU over 90 min (patient HL).

Blood for steady-state drug concentration measurements was drawn during the last hour of the infusion. The kinetic data of the prolonged s.c. infusion are summarised in Table III. Patient RF developed a painless local skin pigmentation and later desquamation. However, a similiar skin reaction was observed at the catheter site (arm) following the next intravenous infusion.

### Discussion

5-FU is an important component in the treatment of a variety of solid tumours. In colorectal cancer 5-day high-dose or prolonged (several weeks) continuous low-dose infusion have shown higher response rates and were better tolerated than standard 5-day monthly bolus regimen (Seifert et al., 1975; Lokich et al., 1989). In head and neck cancer the most potent drug combinations include continuous 5-FU (Kish et al., 1984; Dreyfuss et al., 1990) which was superior to bolus

**Table III** Pharmacokinetic parameters after s.c. or i.v. infusion of indicated 5-FU dose over 24 h

| Patient | Daily dose | Steady-state concentration (ng ml$^{-1}$) s.c. | i.v. |
|---|---|---|---|
| BM | 1500 mg | 166 | 112 |
| MH | 1750 mg | 176 | 190 |
| RF | 1500 mg | 403 | 531 |

**Table II** Pharmacokinetic parameters after s.c. and i.v. infusion of 250 mg 5-FU over 90 min

| Patient | Area under the curve (ng h ml$^{-1}$) s.c. | i.v. | Bioavailability | Peak concentration (ng ml$^{-1}$) s.c. | i.v. | Steady-state concentration (ng ml$^{-1}$) |
|---|---|---|---|---|---|---|
| AA | 234 | 285 | 0.82 | 163 | 296 | 150 |
| BA | 398 | 600 | 0.66 | 215 | 501 | 203 |
| BH | 715 | 557 | 1.28 | 523 | 615 | |
| BeH | * | 777 | * | * | 567 | |
| BM | 892 | 928 | 0.96 | 750 | 757 | |
| BV | 1040 | 1070 | 0.97 | 683 | 855 | |
| HL | 582 | 795 | 0.73 | 575 | 587 | |
| ME | 1075 | 1009 | 1.07 | 682 | 840 | |
| RF | 1070 | * | * | 545 | * | |
| SA | 306 | 511 | 0.60 | 204 | 465 | 193 |

*not done.

administration (Kish *et al.*, 1985). Finally, a fraction of breast cancer patients refractory to 5-FU-containing combinations still responds to a prolonged low-dose infusion of 5-FU alone (Huan *et al.*, 1989). In most of these situations the therapy has mainly palliative intent. Costs, the complications of a permanent catheter, and in some situations the need for hospitalisation are disadvantages of prolonged i.v. infusion. However, these problems are circumventable by s.c. drug administration (Cerny *et al.*, 1990; Cerny *et al.*, 1991). Not surprisingly, ambulatory treatment is preferred by most patients (Vokes *et al.*, 1989; Cerny *et al.*, 1990; Cerny *et al.*, 1991).

Our results show that s.c. 5-FU has an almost complete bioavailability (mean: 89%). For the calculation of the bioavailability a constant clearance has been assumed. However, as pointed out earlier it is known that the clearance of 5-FU is saturable (Collins *et al.*, 1980). We chose a relatively high dose rate of 5-FU (250 mg /1.5 h) to increase the chance to be well above the detection limit also in case of poor s.c. absorption. Thus it is possible that the actual clearance of the s.c. infusion was higher compared to the i.v. infusion. The results from three patients which received a 24-h s.c. infusion seem to show a good absorption from the s.c. administration site also over an extended time period.

Flaws of our pharmacokinetic analysis are the short infusion duration without reaching the steady-state concentration in most of the patients and irregularities of the pump flow, which lead to the premature run out of the infusion in some cases. However, the goal of this study, to assess bioavailability and feasibility of s.c. 5-FU, should not have been hampered by these factors.

The tolerability of the s.c. infusion was satisfactory. One patient (RF) which reacted to the 24-h infusion with painless local skin pigmentation and desquamation, showed the same reaction at the forearm after intravenous infusion. Thus, this side effect cannot be attributed to the mode of 5-FU administration.

We conclude that s.c. 5-FU is well tolerated and has a good bioavailability. Before accepting this way of administration as equivalent to the i.v. infusion, it should be shown that these favourable characteristics are maintained over a prolonged treatment period.

The authors wish to express their thanks to Liselotte Dietrich and all the nurses involved for their expertise, initiative, and dedicated patient care.

### References

AL-SARRAF, M. (1988). Head and neck cancer: chemotherapy concepts. *Semin. Oncol.*, **15**, 70–85.

BRUCKNER, H.W. & MOTWANI, B.T. (1991). Chemotherapy of advanced cancer of the colon and rectum. *Semin. Oncol.*, **18**, 443–461.

CERNY, T., GRAF, A., ROHNER, P., ZEUGIN, T., BRUNNER, K.W. & KÜPFER, A. (1991). Subcutaneous continuous infusion of ifosfamide and cyclophosphamide in ambulatory cancer patients: bioavailability and feasibility. *J. Cancer Res. Clin. Oncol.*, **117**, 148–153 (Supplement 4).

CERNY, T., KÜPFER, A., ZEUGIN, T. & BRUNNER, K.W. (1990). Bioavailability of subcutaneous ifosfamide and feasibility of continuous outpatient application in cancer patients. *Ann. Oncol.*, **1**, 365–368.

COLLINS, J.M., DEDRICK, R.L., KING, F.G., SPEYER, J.L. & MYERS, C.E. (1980). Nonlinear pharmacokinetic models for 5-fluorouracil in man: intravenous and intraperitoneal routes. *Clin. Pharmacol. Ther.*, **28**, 235–246.

DREYFUSS, A.I., CLARK, J.R., WRIGHT, J.E., NORRIS, C.M. & FREI, E. III (1990). Continuous infusion high-dose leucovorin with 5-fluorouracil and cisplatin for untreated stage IV carcinoma of the head and neck. *Ann. Intern. Med.*, **112**, 167–172.

HANSEN, R.M., QUEBBEMAN, E. & ANDERSON, T. (1989). 5-fluorouracil by protracted infusion. *Oncology*, **46**, 245–250.

HUAN, S., PAZDUR, R., SINGHAKOWINTA, A., SAMAL, B. & VAITKEVICIUS, V.K. (1989). Low-dose continuous infusion 5-fluorouracil: evaluation in advanced breast carcinoma. *Cancer*, **63**, 419–422.

KISH, J.A., ENSLEY, J.F., JACOBS, J., CUMMINGS, G. & AL-SARRAF, M. (1985). A randomized trial of cisplatin (CACP) + 5-fluorouracil (5-FU) infusion and CACP + 5-FU bolus for recurrent and advanced squamous cell carcinoma of the head and neck. *Cancer*, **56**, 2740–2744.

KISH, J.A., WEAVER, A., JACOBS, J., CUMMINGS, G. & AL-SARRAF, M. (1984). Cisplatin and 5-fluorouracil infusion in patients with recurrent and disseminated epidermoid cancer of the head and neck. *Cancer*, **53**, 1819–1824.

LOKICH, J.J., AHLGREN, J.D., GULLO, J.J., PHILIPS, J.A. & FRYER, J.G. (1989). A prospective randomized comparison of continuous infusion fluorouracil with conventional bolus schedule in metastatic colorectal carcinoma: a Mid-Atlantic Oncology Program Study. *J. Clin. Oncol.*, **7**, 425–432.

MACDONALD, J.S. (1989). Continuous low-dose infusion of fluorouracil: is the benefit worth the cost? *J. Clin. Oncol.*, **7**, 412–414.

SEIFERT, P., BAKER, L.H., REED, M.L. & VAITKEVICIUS, V.K. (1975). Comparison of continuously infused 5-fluorouracil with bolus injection in treatment of patients with colorectal adenocarcinoma. *Cancer*, **36**, 123–128.

STETSON, P.L., SHUKLA, U.A. & ENSMINGER, W.D. (1985). Sensitive high-performance liquid chromatographic method for the determination of 5-fluorouracil in plasma. *J. Chromatogr.*, **344**, 385–390.

VOKES, E.E., SCHILSKY, R.L. & CHOI, K.E. (1989). A randomized study of inpatient versus outpatient continuous infusion chemotherapy for patients with locally advanced head and neck cancer. *Cancer*, **63**, 30–36.